# Radiogenomics nomogram based on MRI and microRNAs to predict microvascular invasion of hepatocellular carcinoma

**DOI:** 10.3389/fonc.2024.1371432

**Published:** 2024-07-11

**Authors:** Guangchao Hu, Jianyi Qu, Jie Gao, Yuqian Chen, Fang Wang, Haicheng Zhang, Han Zhang, Xuefeng Wang, Heng Ma, Haizhu Xie, Cong Xu, Naixuan Li, Qianqian Zhang

**Affiliations:** ^1^ Department of Radiology, Qingdao Municipal Hospital, Qingdao, Shandong, China; ^2^ Department of Radiology, Zhongshan Hospital, Fudan University, Shanghai, China; ^3^ Department of Hepatobiliary Surgery, Yantai Yuhuangding Hospital, Qingdao University, Yantai, Shandong, China; ^4^ School of Information and Electronic Engineering, Shandong Technology and Business University, Yantai, Shandong, China; ^5^ Department of Pathology, Yantai Yuhuangding Hospital, Qingdao University, Yantai, Shandong, China; ^6^ Department of Radiology, Yantai Yuhuangding Hospital, Qingdao University, Yantai, Shandong, China; ^7^ Department of Physical Examination Center, Yantai Yuhuangding Hospital, Qingdao University, Yantai, Shandong, China; ^8^ Department of Interventional Vascular Surgery, Yantai Affiliated Hospital of Binzhou Medical University, Yantai, Shandong, China

**Keywords:** hepatocellular carcinoma (HCC), microvascular invasion (MVI), radiogenomics, nomogram, MicroRNAs, dynamic contrast-enhanced magnetic resonance imaging (DCE-MRI)

## Abstract

**Purpose:**

This study aimed to develop and validate a radiogenomics nomogram for predicting microvascular invasion (MVI) in hepatocellular carcinoma (HCC) on the basis of MRI and microRNAs (miRNAs).

**Materials and methods:**

This cohort study included 168 patients (training cohort: n = 116; validation cohort: n = 52) with pathologically confirmed HCC, who underwent preoperative MRI and plasma miRNA examination. Univariate and multivariate logistic regressions were used to identify independent risk factors associated with MVI. These risk factors were used to produce a nomogram. The performance of the nomogram was evaluated by receiver operating characteristic curve (ROC) analysis, sensitivity, specificity, accuracy, and F1-score. Decision curve analysis was performed to determine whether the nomogram was clinically useful.

**Results:**

The independent risk factors for MVI were maximum tumor length, rad-score, and miRNA-21 (all P < 0.001). The sensitivity, specificity, accuracy, and F1-score of the nomogram in the validation cohort were 0.970, 0.722, 0.884, and 0.916, respectively. The AUC of the nomogram was 0.900 (95% CI: 0.808–0.992) in the validation cohort, higher than that of any other single factor model (maximum tumor length, rad-score, and miRNA-21).

**Conclusion:**

The radiogenomics nomogram shows satisfactory predictive performance in predicting MVI in HCC and provides a feasible and practical reference for tumor treatment decisions.

## Introduction

Hepatocellular carcinoma (HCC) is a malignancy with third highest world mortality rate (8.3%), after lung cancer (18%) and colorectal cancer (9.4%) (1). The main treatment of HCC is surgical resection, but recurrence is common, with a five-year recurrence rate of up to 40%–70%. The 5-year survival rate is only 18% (2). Microvascular invasion (MVI) is one of the most important prognostic factors for HCC after surgical treatment, and it has been established as a risk factor for early recurrence and poor outcome. The term MVI refers to the cancer cell nests that are established within the lining of blood vessels by microscopic endothelial cells. It is considered as a sign of strong tumor invasion ability, and it could only be diagnosed through pathology at present. MVI-positive patients often require improved prognosis by expanding surgical margins, and patients with microvascular infiltration are also considered unsuitable for liver transplantation (3–5). Therefore, developing a method for non-invasive prediction of microvascular invasion is necessary to guide the treatment of HCC.

The imaging characteristics of HCC, such as a non-smooth tumor margin, arterial peritumoral enhancement, and peritumoral hypo-intensity on hepatobiliary phase imaging (HBP), have been confirmed to be noninvasive imaging biomarkers for MVI prediction (4–7). However, such qualitative studies are vulnerable to subjective factors, image quality, and interobserver variation. Therefore, more objective quantitative methods are needed to predict MVI. In 2012, Lambin et al. (8) proposed the concept of radiomics, where medical images are converted into useful data by using high-throughput quantitative features to predict the disease treatment efficacy and prognosis. Using radiomics to predict MVI in HCC is a major research area in recent years. Many studies with satisfactory results have been conducted (9–11). Xu’s (10) study has achieved predicted satisfactory results by developing a radiomics nomogram model on the basis of computed tomography (CT). MRI examination technology has the advantage of multimodal/multisequence imaging and high soft-tissue resolution. Theoretically MRI multicolumn multimodal imaging provides more characteristic elements. Therefore, in the present study, predictive models based on radiomic features in MRI were developed for predicting MVI.

MicroRNAs (miRNAs) are a kind of endogenous, non-coding RNAs. Thousands of miRNAs play a role in regulating various molecular biological processes by inhibiting the translation of different messenger RNAs (mRNAs) in the cell (12). A dysregulation of miRNAs is often associated with malignancy, and it regulates the proliferation, migration, invasion, and development of tumors in HCC by promoting or suppressing them (13, 14). Previous studies have shown that combining radiomics and genomics could remarkably improve the performance of predictive models (15, 16). Zhou (17) et al. screened 7 plasma miRNAs (miRNAs) out of 723 HCC-associated miRNAs (miR-122, miR-192, miR-21, miR-223, miR-26a, miR-27a, miR-801), which had high diagnostic performance in the early diagnosis of hepatocellular carcinoma.Therefore, we extracted these 7 mi-RNAs from the patients’ plasma, but among them, miR-192 and miR-801 had large differences in expression, and the data were not stable enough to be screened out, so only 5 mi-RNAs (miR-122, miR-21, miR-223, miR-26a, miR-27a) were included in the analysis. Therefore, we measured these miRNAs and explored their relationship with HCC microvascular invasion, and combined them with radiomics to explore the performance of the joint model. Moreover, routine laboratory tests for HCC and radiological characteristics based on MRI were added; the independent risk factors for MVI were determined through multivariate logistic regression, combined radiomics, genomics, and clinico-radiological factors; predictive models were established; and the performance of these models was verified.

This study aimed to develop and validate a radiogenomics nomogram model for preoperative prediction of MVI in HCC. The nomogram is helpful for clinicians to assist in determining individual therapeutic strategies for patients with HCC.

## Materials and methods

### Patients

This retrospective study was approved by the institutional review board, with a waiver for patient informed consent. We included all patients who underwent preoperative MRI and plasma mi-RNAs between December 2018 and November 2021. The inclusion criteria were as follows: (a) all patients who underwent radical hepatectomy with postoperative pathologic confirmation of hepatocellular carcinoma and complete clinical data;(b) MRI examination and plasma miRNA testing within two weeks prior to surgery; (c) Histopathology report containing a complete description of hepatocellular carcinoma (tumor size, number, MVI status and category, etc.); (d) The images were free of artifacts, sequence loss, and high image quality, meeting the basic requirements for image segmentation. The exclusion criteria were as follows: (a) the patient underwent any form of anticancer treatment (surgery, drugs, etc.) before surgery;(b) vascular or vascular invasion or the presence of distant metastasis was detected by the naked eye in preoperative imaging;(c) combined with other primary tumors.

**Figure 1:**
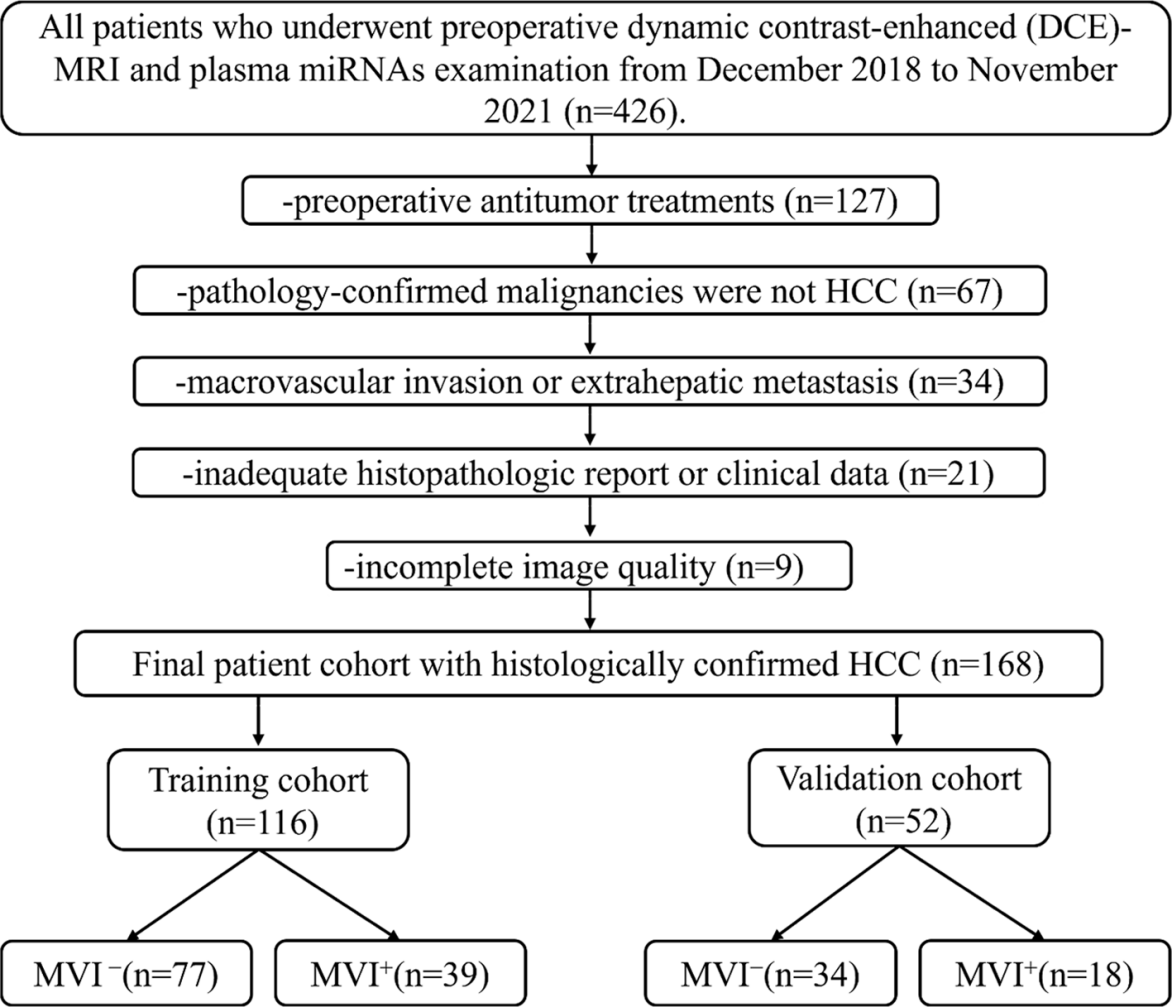
Flowchart of patient enrollment and grouping.

Then, 168 patients (142 males and 26 females) comprised the final cohort. The included patients were divided into training (n = 116; 100 males and 16 females) and validation cohorts (n = 52; 43 males and 9 females), with a ratio of 7:3. The flowchart of patient enrollment and grouping in **Figure 1**. All patients received routine laboratory tests and plasma miRNA examinations prior to curative resection. Further information on the patients is available in **Table 1**.

**Table 1:** Comparisons of patient characteristics in training and validation cohorts.

Characteristics	Training cohort			Validation cohort		
	MVI^+^ (n=39)	MVI^−^ (n=77)	*P*	MVI^+^ (n=18)	MVI^−^ (n=34)	*P*
Age (years)	58.97 ± 8.49	59.48 ± 8.90	0.769	58.89 ± 10.22	56.03 ± 11.33	0.375
Sex Male Female	29 (74.36%) 10 (25.64%)	33 (84.62%) 6 (15.38%)	0.726	13 (72,22%) 5 (27.78%)	29 (85.29%) 5 (14.71%)	0.304
HBV Present Absent	38 (97.44%) 1 (2.56%)	73 (94.81%) 4 (5.19%)	0.514	18 (100.0%) 0(0.00%)	32 (94.12%) 2 (5.88%)	0.160
AFP (ng/mL) ≤ 20 20–400 >400	19 (48.7%) 8 (20.5%) 12 (30.8%)	39 (50.6%) 22 (28.6%) 16 (20.8%)	0.079	11 (61.1%) 2 (11.1%) 5 (27.8%)	15 (44.1%) 12 (35.3%) 7 (20.6%)	0.383
ALT (µ/l) ≤ 40 >40	25 (64.1%) 14 (36.9%)	55 (71.4%) 22 (28.6%)	0.318	10 (55.6%) 8 (44.4%)	23 (67.6%) 11 (32.4%)	0.372
AST (µ/l) ≤ 35 >35	22 (56.4%) 17 (43.6%)	42 (45.5%) 35 (54.5%)	0.364	10 (55.6%) 8 (44.4%)	22 (64.7%) 12 (35.3%)	0.415
TBIL (µmol/l) ≤ 20 >20	26 (66.7%) 13 (33.3%)	53 (68.8%) 24 (31.2%)	0.379	12 (66.7%) 6 (33.3%)	22 (64.7%) 12 (35.3%)	0.418
ALB (g/l) ≤ 40 >40	17 (43.6%) 22 (56.4%)	42 (54.5%) 35 (45.4%)	0.635	9 (50.0%) 9 (50.0%)	19 (55.9%) 15 (44.1%)	0.869
PIVKA-II (mAu/mL) ≤ 40 >40	4 (10.3%) 35 (89.7%)	11 (14.3%) 66 (85.7%)	0.910	4 (22.2%) 14 (77.8%)	5 (14.7%) 29 (85.3%)	0.144
PT (s) ≤ 14 >14	35 (89.7%) 4 (10.3%)	68 (88.3%) 9 (11.7%)	0.941	17 (94.4%) 1 (5.6%)	29 (85.3%) 5 (14.7%)	0.679
INR ≤ 1.0 >1.0	18 (46.2%) 21 (53.8%)	18 (23.4%) 59 (76.6%)	0.382	8 (44.4) % 10 (55.6%)	8 (23.5%) 26 (76.5%)	0.376
MiRNA-21	29.60 ± 1.40	31.32 ± 0.85	< 0.001	29.18 ± 1.19	31.13 ± 0.76	< 0.001
MiRNA-26a	32.08 ± 1.67	30.27 ± 1.69	< 0.001	32.32 ± 1.53	30.40 ± 1.60	< 0.001
MiRNA-27a	27.35 ± 2.04	28.51 ± 0.95	0.002	26.50 ± 1.97	28.46 ± 1.03	< 0.001
MiRNA-122	28.96 ± 1.62	29.89 ± 0.95	0.001	28.35 ± 1.48	29.81 ± 0.97	< 0.001
MiRNA-223	31.88 ± 1.50	31.13 ± 1.06	0.001	32.46 ± 1.30	31.25 ± 1.07	< 0.001
Maximum tumor length	6.43 ± 3.36	5.16 ± 2.58	0.044	6.11 ± 2.88	5.24 ± 3.05	0.320
Tumor margin Smooth Non-smooth	3 (7.7%) 36 (92.3%)	23 (29.9%) 54 (70.1%)	0.007	1 (5.6%) 17 (94.4%)	12 (35.3%) 22 (64.7%)	0.043
Number =1 >1	36 (92.3%) 3 (7.7%)	68 (88.3%) 9 (11.7%)	0.730	16 (88.9%) 2 (11.1%)	31 (91.2%) 3 (8.8%)	1.000
Enhancement pattern Typical Atypical	35 (89.7%) 4 (10.3%)	73 (94.8%) 4 (5.2%)	0.530	2 (11.1%) 16 (88.9%)	32 (94.1%) 2 (5.9%)	0.900
Radiologic capsule Present Absent	7 (17.9%) 32 (82.1%)	40 (51.9%) 37 (48.1%)	< 0.001	3 (16.7%) 15 (83.3%)	20 (58.8%) 14 (41.2%)	0.004
Arterial peritumoral enhancement Present Absent	36 (92.3%) 3 (7.7%)	44 (57.1%) 33 (42.9%)	< 0.001	17 (94.4%) 1 (5.6%)	15 (44.1%) 19 (55.9%)	0.001
Intratumor necrosis/hemorrhage Present Absent	33 (84.6%) 6 (15.4%)	45 (58.4%) 32 (41.6%)	0.005	17 (94.4%) 1 (5.6%)	19 (55.9%) 15 (44.1%)	0.011

HBV, hepatitis B virus; AFP, serum alpha-fetoprotein; ALT, alanine aminotransferase; AST, aspartate amino transferase; TBIL, total bilirubin; ALB, albumin; PIVKA-II, protein induced by vitamin K absence or antagonist-II; PT, prothrombin time; INR, international normalized ratio; MVI, microvascular invasion.

### Histopathological examination

Seven-point baseline sampling method was used to take 1:1 samples at the junction between the cancer and the paracancerous liver tissues at the clock positions of twelve, three, six, and nine points of the tumor. At least one piece of tissue was taken inside the tumor, and one piece of liver tissue was taken ≤ 1 cm (near paracancerous) and > 1 cm (distal paracancerous) from the tumor margin (18). Histopathological features (tumor size, number, MVI status, and category) were consistently assessed by two experienced abdominal pathologists.

### Collection of plasma samples and miRNA extraction

Venous blood samples were collected from all patients with HCC prior to any means of processing. Before the sample collection was conducted, a written consent was obtained for each patient to donate a sample for the purpose of the study. For specific steps on collection of plasma samples and miRNA extraction, please refer to the **Supplementary Materials 1**.

### MRI examination

MRI examinations were conducted using a GE DISCOVERY 750W 3.0 T MRI scanner, with axial in-phase and opposed-phase T1 weighted imaging (T1WI), axial T2-weighted imaging with fat suppression (T2WI-FS), diffusion-weighted imaging (DWI), and DCE-MRI (dynamic contrast-enhanced magnetic resonance imaging) sequences for all patients. Please refer to **Supplementary Material 2** for specific MRI parameters.

### Analysis of radiological characteristics

The radiological characteristics were independently evaluated by two abdominal radiologists A (RA) and B (RB), with 7 and 15 years of experience, respectively. If any differences occurred, senior radiologist C (RC) with 20 years of experience would join the discussion to reach a consensus. All radiologists were aware that the lesions were HCCs but blinded to all other laboratory and histopathological information. The largest tumor was used to analyze patients with multiple tumors. The evaluation was based on the Liver Imaging Reporting and Data System (LI-RADS version 2018) (19), and the important morphological features reported in the relevant literature (7). The qualitative features of the images were assessed refer to **Supplementary Material 3**.

### Analysis of radiomics

#### Image segmentation

HCC image segmentation was performed by RA and RB with the use of three-dimensional (3D) slicer software (version 5.0.2). The volumes of interest (VOIs) were delineated in the axial T2WI-FS, DWI (with b value of 800 s/mm^2^), AP, PP, and DP images. For assessment of the reproducibility and reliability of image segmentation, images of 30 randomly selected patients were first segmented by RA and RB separately. Then, 30 patients were re-segmented by RB after 2 weeks, and the images of the remaining patients were segmented by RA. The segmentation results were validated by RC.

#### Radiomics feature extraction, selection, and signature building

A total of 7045 radiomic features were extracted from each segmented lesion using the SlicerRadiomics plugin in 3DSlicer. Python (version 2.7.18) was used for radiological feature selection. These features included shape, first-order histogram features and texture features. The 1856 features with intra- and inter-correlation coefficients (ICCs) values less than 0.8 were firstly excluded, and the remaining features were initially screened by SelectKBest. The remaining features were selected by least absolute shrinkage and selection operator (LASSO) algorithms. The features of the LASSO regression result in which the corresponding coefficients with non-zero were retained. 10-fold cross-validation was performed to select the optimal α value, and the coefficients of the corresponding radiomics features were obtained at the same time. The radiomic feature score (rad-score) reflecting the MVI was calculated for each patient by using a linear combination of the selected features weighted with the respective coefficients.

#### Model construction, evaluation, and comparison

**Figure 2:**
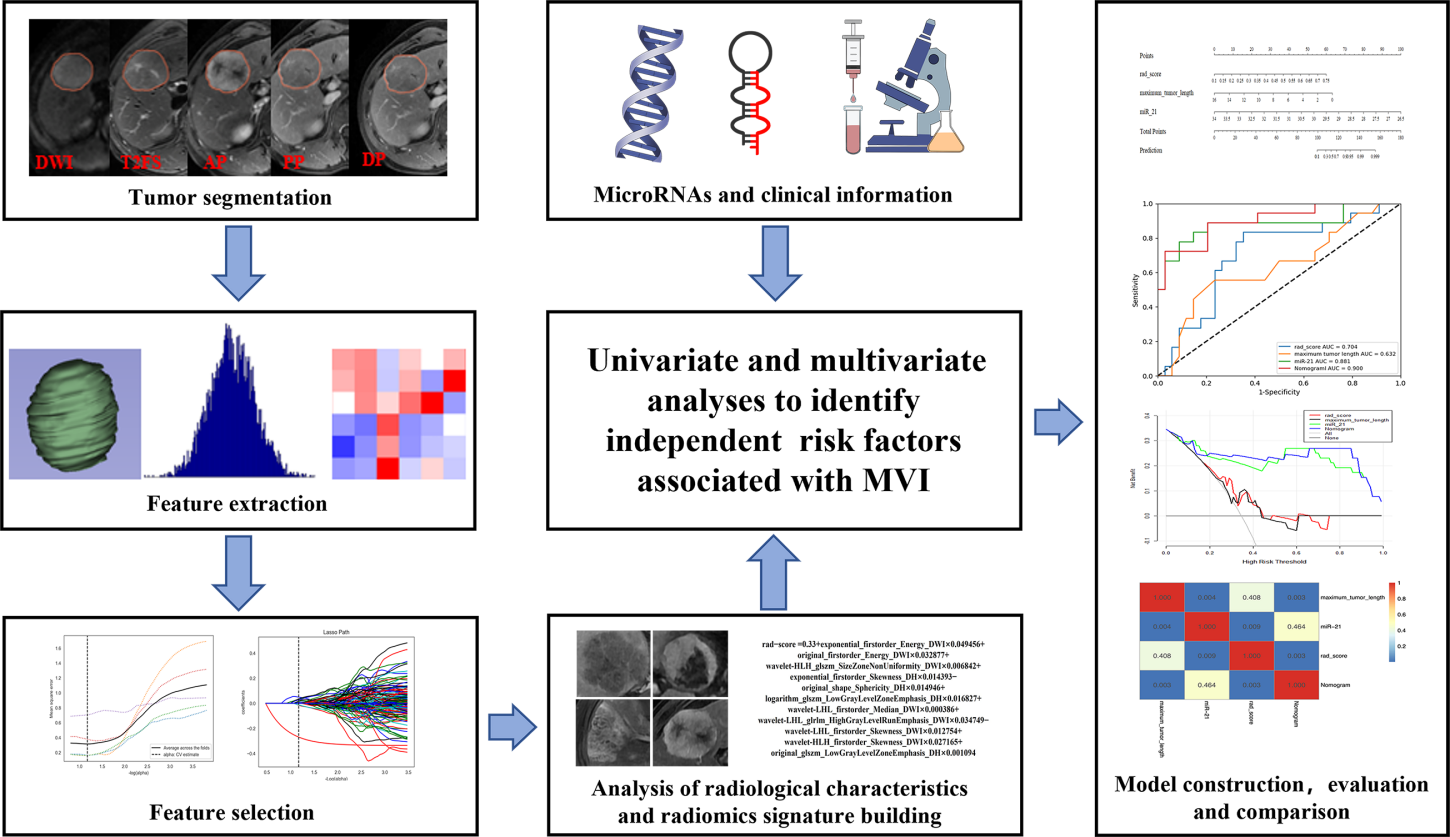
Flowchart showing the radiogenomics analysis for MVI prediction. ROI segmentation was performed on axial MR images, and then radiomic features were extracted and selected. Next, the radiomic score was calculated for each patient by using a linear combination of selected features weighted with the respective coefficients. The radiological characteristics, miRNAs, and clinical information were also collected. Finally, all variables were screened by univariate and multivariate logistic regression analyses to identify the independent risk factors for MVI, which were used to construct the nomogram. The nomogram was evaluated with ROC curve and decision curve. Delong test was used to compare area under the curves (AUCs) from different models.

All variables (laboratory tests, miRNA, radiological characteristics, and radiation scores) were first screened by univariate analysis, and then independent risk factors for MVI were determined by stepwise backward regression with the principle of minimum AIC (Akaike information criterion) value by multivariate logistic regression analysis. All the independent risk factors were used separately to build the corresponding prediction models and construct the nomogram. The ROC curves were plotted, the discriminant efficiency of MVI predictions was quantified using AUC, and multiple comparisons between different models were carried out by Delong test. The 95% CI of AUCs, sensitivity, specificity, and accuracy were also calculated. F1-score was used to evaluate a binary classification model with unbalanced data samples. The clinical utility of the nomogram was evaluated using decision curve analysis, which quantifies the net benefit to the overall cohort at different threshold probabilities (20). The process of the present study is illustrated in **Figure 2**.

### Statistical analysis

Statistical analysis was performed using R software (version 3.4.1). Continuous variables were expressed as mean ± standard deviation. The categorical variables were presented as percentages. Kolmogorov–Smirnov tests were used to evaluate the distribution’s normality. For identification of variables that differed significantly between the training and validation cohorts, Student’s t test was used to compare the quantitative data, and Chi-square test or Fisher’s exact test was used to compare the qualitative data. The reproducibility of the feature extraction was assessed by calculating the intra- and inter-correlation coefficients (ICCs), and ICCs > 0.80 were considered to have good reproducibility. The Hosmer–Lemeshow’s goodness-of-fit test was used to evaluate whether the model’s predicted probabilities fitted the actual probabilities. The sensitivity, specificity, and accuracy were calculated by confusion matrix in accordance with the cutoff value that maximized the Youden index. Statistical significance was set at *P* < 0.05.

## Results

### Clinico-radiological characteristics and MVI prediction factors

A comparison of the clinico-radiological characteristics is shown in **Table 1**. Among the 168 patients with HCC, MVI was diagnosed in the resected tissue of 57 patients. The comparison between the training and validation cohorts was not statistically different in terms of age, gender, AFP, and other clinical indicators (*P* = 0.144–0.941). All five miRNAs significantly differed between MVI^+^ and MVI^−^ in the training and validation cohorts (*P* < 0.05). The patients with MVI^+^ and MVI^−^ also showed significantly different imaging characteristics (tumor margin, radiologic capsule, arterial peritumoral enhancement, and intratumor necrosis/hemorrhage) (*P* < 0.05). No significant differences were found in the tumor number and enhancement pattern between MVI^+^ and MVI^−^ in either the training cohort or the validation cohort (*P* = 0.530–1.000).

The univariate analysis showed that five imaging features (maximum tumor length, tumor margin, radiologic capsule, peri-arterial tumor enhancement, and presence of hemorrhage and necrosis) and the five kinds of miRNAs (miR-21, miR-26a, miR-27a, miR-122, and miR-223) were significantly associated with MVI (*P* < 0.05). In the multivariate analysis, maximum tumor length and miR-21 were found to be independent predictors of MVI. The specific information is shown in **Table 2**.

**Table 2:** Univariate and multivariate analysis to identify risk factors associated with MVI in the training cohort.

Variables	Univariate analysis		Multivariate analysis	
	OR (95% CI)	*P*	OR (95% CI)	*P*
Sex	0.95 (0.74–1.23)	0.726	NA	NA
Age	1.00 (0.99–1.01)	0.769	NA	NA
HBV	1.15 (0.75–1.77)	0.514	NA	NA
AFP	1.00 (0.99–1.01)	0.198	NA	NA
PIVKA-II	1.00 (0.99–1.00)	0.906	NA	NA
ALT	0.99 (0.99–1.00)	0.318	NA	NA
AST	0.99 (0.99–1.00)	0.364	NA	NA
TB	0.98 (0.99–1.02)	0.379	NA	NA
ALB	1.01 (0.99–1.13)	0.641	NA	NA
PT	0.98 (0.94–1.06)	0.941	NA	NA
INR	0.96 (0.87–1.05)	0.382	NA	NA
MiRNA-21	0.81 (0.76–0.85)	< 0.001	0.73 (0.66–0.78)	< 0.001
MiRNA-26a	1.12 (1.08–1.17)	< 0.001	1.05 (0.99–1.11)	0.065
MiRNA-27a	0.89 (0.84–0.94)	< 0.001	0.95 (0.81–1.11)	0.518
MiRNA-122	0.88 (0.83–0.94)	0.002	1.04 (0.74–1.44)	0.840
MiRNA-223	1.11 (1.04–1.19)	< 0.001	0.86 (0.68–1.09)	0.220
Maximum tumor length	1.05 (1.02–1.07)	< 0.001	0.90 (0.87–0.93)	< 0.001
Tumor margin	1.33 (1.09–1.07)	< 0.001	0.99 (0.83–1.18)	0.478
Number	0.91 (0.68–1.21)	0.509	NA	NA
Enhancement pattern	0.84 (0.60–1.18)	0.314	NA	NA
Radiologic capsule	0.73 (0.62–0.86)	< 0.001	0.92 (0.79–1.07)	0.788
Arterial peritumoral enhancement	1.44 (1.21–1.71)	< 0.001	1.10 (0.93–1.29)	0.329
Intratumor necrosis/hemorrhage	1.30 (1.09–1.56)	< 0.001	0.93 (0.81–1.08)	0.915
Rad-score	9.33 (4.65–18.74)	< 0.001	7.92 (3.78–16.6)	< 0.001

HBV, hepatitis B virus; AFP, serum alpha-fetoprotein; ALT, alanine aminotransferase; AST, aspartate amino transferase; TBIL, total bilirubin; ALB, albumin; PIVKA-II, protein induced by vitamin K absence or antagonist-II; PT, prothrombin time; INR, international normalized ratio; OR, odds ratio; CI, confidence interval. NA, not available.

### Feature selection and radiomics signature building

Radiomics features were downscaled by SelectKBest and LASSO, resulting in a final selection of 11 features, all of which were derived from DWI and DP sequences. The ICCs ranged from 0.856 to 0.989 for the intra-observers and from 0.843 to 0.982 for the inter-observers. These values demonstrated the high reliability of the measurements taken by the observers. A linear combination of the selected features, weighted by their respective logistic regression coefficients, was used to generate the rad-score (risk score reflecting the probability of MVI). This score was used to calculate each selected VOI as follows:

*rad-score = 0.33 + exponential_firstorder_Energy_DWI × 0.049456 + original_firstorder_Energy_DWI × 0.032877 +*

*wavelet-HLH_glszm_SizeZoneNonUniformity_DWI × 0.006842 + exponential_firstorder_Skewness_DH × 0.014393 −*

*original_shape_Sphericity_DH × 0.014946 +*

*logarithm_glszm_LowGrayLevelZoneEmphasis_DH × 0.016827 +*

*wavelet-LHL_firstorder_Median_DWI × 0.000386 +*

*wavelet-LHL_glrlm_HighGrayLevelRunEmphasis_DWI × 0.034749 −*

*wavelet-LHL_firstorder_Skewness_DWI × 0.012754 +*

*wavelet-HLH_firstorder_Skewness_DWI × 0.027165 + original_glszm_LowGrayLevelZoneEmphasis_DH × 0.001094*

The univariate and multifactorial regression analyses showed that the rad-score is an independent risk factor for MVI (**Table 2**).

### Model construction and evaluation

**Figure 3:**
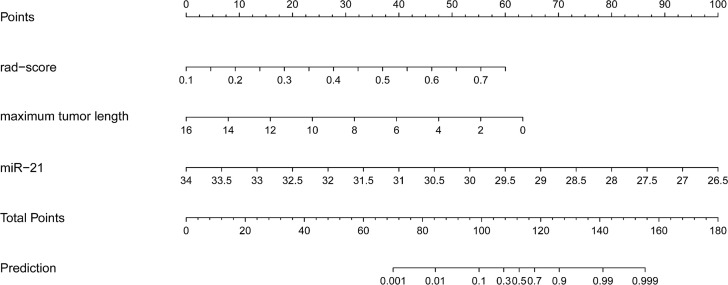
Radiogenomics nomogram for predicting MVI in hepatocellular carcinoma. (1) Factors in the prediction model: maximum tumor length, rad-score, and miRNA-21, the scale on the line segment corresponding to each factor represents the range of values of the factor, and the length of the line segment represents the contribution of the factor to the probability of occurrence of the outcome variable. (2) Points and total pionts: individual points represent the individual scores corresponding to each factor at different ranges of values, and the total points represents the sum of the individual scores corresponding to all the factors at different ranges of values. (3) Prediction: the scale value corresponding to the total points indicates the risk of microvascular invasion in patients with hepatocellular carcinoma.

The independent predictive factors of MVI, which were maximum tumor length, miR-21, and rad-score, were identified by univariate and multivariate logistic regression methods. The MVI prediction model incorporated these three independent risk factors to develop a nomogram prediction model (**Figure 3**).

**Figure 4:**
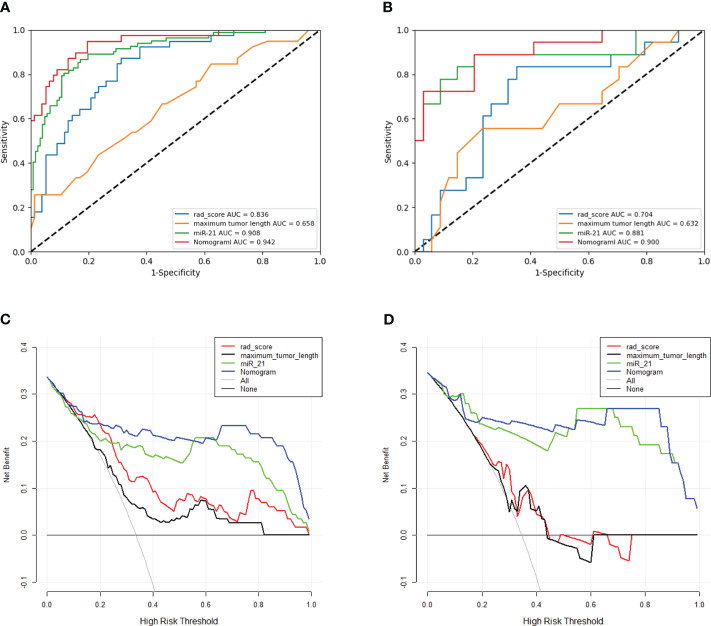
**(A, B)** ROC curves for different models in the training and validation cohorts. The ROC graph is a curve that reflects the relationship between sensitivity and specificity. According to the position of the curve, the whole graph is divided into two parts, the area below the curve is called AUC (Area Under Curve), which is used to indicate the prediction accuracy, the higher the value of AUC, that is, the larger the area under the curve, indicating that the prediction accuracy is higher. The closer the curve is to the upper left corner, the higher the prediction accuracy. Panels **A**, **B** represent the prediction values of different models for the training cohort and validation cohort, respectively. **(C, D)** Clinical decision curves for different models in the training and validation cohorts. Clinical utility is evaluated in terms of Decision curve analysis (DCA), which reflects the ability of a model to benefit patients by influencing clinical decisions.A good model should have a high net benefit value at the threshold required by its clinical question. The net benefit of predicting the decision curve for the nomogram and miR-21 model was higher than that for other models when the threshold probability was > 0%. This finding suggested that the nomogram and miR-21 models could achieve satisfactory net clinical benefits.

The three single-factor models of tumor maximum length, miR-21, and rad-score reached AUC values of 0.658 (95% CI: 0.551–0.764), 0.907 (95% CI 0.866–0.949), and 0.836 (95% CI: 0.763–0.909) in the training cohort, respectively, and 0.632 (95% CI: 0.465–0.799), 0.881 (95% CI: 0.763–0.998), and 0.704 (95% CI: 0.551–0.857) in the validation cohort, respectively. The nomogram model had an AUC of 0.900 (95% CI 0.808–0.992) in the validation cohort, with sensitivity, specificity, accuracy, and F1-score of 0.970, 0.722, 0.884, and 0.916, respectively (**Figures 4A, B**, **Table 3**). The Hosmer–Lemeshow’s goodness-of-fit test evaluated the model performance at *P* = 0.55 > 0.05, indicating that the actual value of the prediction model fitted well with the predicted value. The decision curve showed the clinical usefulness of the different models (**Figures 4C, D**). The prediction performance of the nomogram model was satisfactory in the validation cohort, with the decision curve shown in **Figure 4D**. The net benefit of predicting the decision curve for the nomogram and miR-21 model was higher than that for other models when the threshold probability was > 0%. This finding suggested that the nomogram and miR-21 models could achieve satisfactory net clinical benefits.

**Table 3:** Performance of different MVI prediction models.

Models	AUC	Sensitivity	Specificity	Accuracy	F1-score
Maximum tumor length Training cohort Validation cohort	0.658 (0.551–0.764) 0.632 (0.465–0.799)	0.987 0.764	0.256 0.555	0.698 0.692	0.835 0.764
Rad-score Training cohort Validation cohort	0.836 (0.763–0.909) 0.704 (0.551–0.857)	0.688 0.647	0.871 0.833	0.750 0.711	0.785 0.746
MiRNA-21 Training cohort Validation cohort	0.907 (0.866–0.949) 0.881 (0.763–0.998)	0.827 0.911	0.865 0.777	0.843 0.865	0.875 0.898
Nomogram Training cohort Validation cohort	0.942 (0.899–0.985) 0.900 (0.808–0.992)	0.805 0.970	0.948 0.722	0.853 0.884	0.832 0.916

AUC, area under the ROC curve.

### Model comparison

**Figure 5:**
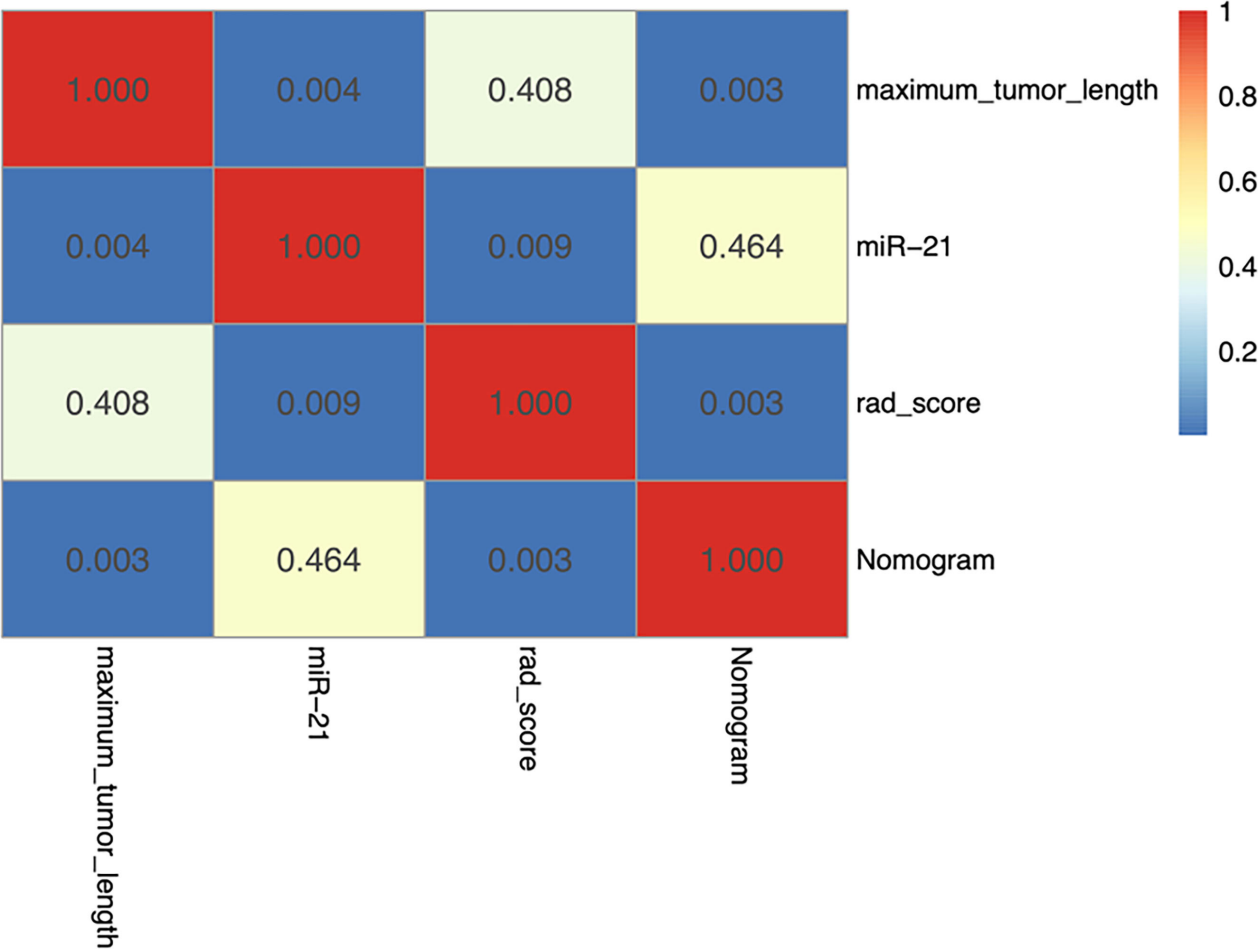
Heatmap showing statistical differences between different models in the validation cohort. The nomogram model outperforms the maximum tumor diameter model and the radiomics scoring model, and the differences are statistically significant (*P* < 0.05). The AUC value of the nomogram model is slightly higher than that of the miR-21 model, although the difference is not statistically significant (*P* = 0.464).

Among the three single-factor models of maximum tumor length, miR-21, and rad-score, the miR-21 model performed best, and the differences with the other two one-factor models were all statistically significant in the validation cohort (miR-21 vs. maximum tumor length: AUC of 0.881 vs. 0.632, *P* = 0.004; miR-21 vs. rad-score: AUC of 0.881 vs. 0.704, *P* = 0.009). The nomogram prediction model outperformed the miR-21 model (AUC of 0.900 vs. 0.881; *P* = 0.464), the rad-score model (AUC of 0.900 vs. 0.704; *P* = 0.003), and the maximum tumor length model (AUC of 0.900 vs. 0.632; *P* = 0.003) in the validation cohort. However, no statistical difference was found between the nomogram model and the miR-21 model (*P* = 0.464), as detailed in **Table 3** and **Figure 5**.

## Discussion

In this study, we have successfully developed and rigorously validated a multi-omics nomogram prediction model, which integrates MRI-derived radiomics, radiological features, and miRNA-based genomics. The resulting radiogenomic nomogram has demonstrated excellent performance in accurately predicting microvascular invasion in HCC, thus providing a non-invasive yet reliable clinical method for preoperative prediction.

The expression of miR-21, miR-27a, and miR-122 in the MVI^+^ group was upregulated compared with that in the MVI^−^ group, whereas the expression of miR-26a and miR-223 was downregulated. These differences were all statistically significant. By contrast, the differences in conventional laboratory indicators, such as AFP, were not statistically significant between the MVI^+^ group and the MVI^−^ group, indicating that the miRNAs extracted in the plasma of patients with HCC were more meaningful in suggesting MVI than the conventional laboratory indicators. Further univariate and multivariate analyses showed that miR-21 had better correlations than other clinical laboratory tests. Studies have shown that miR-21 is one of the most expressed miRNAs in liver diseases, such as nonalcoholic fatty liver disease (21). Ladeiro et al. (22) found that miR-21 was significantly overexpressed in HCC, as compared to benign tumor or non-neoplastic liver tissue. The maladjusted expression of miRNA could be used as a biomarker, and it could be detected in the plasma of patients. Studies have shown that miR-21 plays a role in promoting HCC growth invasion, distant metastasis, and other links (21). Furthermore, this study confirmed that among other miRNAs detected in plasma, only miR-21 emerged as an independent risk factor for MVI in HCC. The miR-21 model surpassed the rad score and maximum tumor length models in predicting MVI, and the differences were statistically significant in the validation cohort (P<0.05). These findings indicate that the miR-21 model performs well in preoperative prediction of MVI in HCC. Conventional clinical laboratory indicators, including tumor markers such as AFP, were all excluded in the univariate analysis due to their relatively poor correlation compared to other variables.

On the basis of the morphological characteristics of MRI, previous meta-analysis studies have found that some of MRI signs were significantly associated with MVI, including larger tumors (> 5 cm), rim arterial enhancement, arterial peritumoral enhancement, non-smooth tumor margin, and multiple lesions (7). The present study incorporated these MR morphological features into the analysis and found that only the maximum tumor length is an independent predictor of MVI in HCC. Increased tumor volume led to increased contact between the tumor and adjacent normal liver tissues, thereby promoting the formation of microvessels.

A total of 7045 features were extracted from five different MRI sequences, and then 11 radiomic features were screened out by SelectKBest and LASSO. Interestingly, these features were all derived from DWI and DP sequences. A previous study (23) found that primary radiomic signatures extracted from delayed-phase sequences were associated with MVI. Zhang et al. (24) compared the performance of different MRI sequences to predict MVI and found the performance of DP to be the best (AUC = 0.806). The present study also found the value of DP to be the best in predicting MVI, which could be explained by the fact that tumors continue to release a large number of angiogenic factors that promote tumor angiogenesis and change tumor perfusion, resulting in the differences between MVI^−^and MVI^+^ being more easily shown in DP sequences (25). DWI also has a satisfactory performance, as confirmed by some previous studies (26, 27). This finding could be explained that the minimum value of the apparent diffusion coefficient of DWI could reflect the densest tumor, the most abundant neovascularization, and the most active tumor proliferation. The hepatobiliary phase of specific contrast agents has been reported to make an important contribution to suggesting MVI (7). It is important to note that when hepatocyte-specific agents are used, DWI is usually scanned after contrast, and the ability and contribution of DWI may differ, which needs to be proven by further research.

After rigorous data analysis and model training, we have successfully developed a nomogram model that comprehensively incorporates various risk factors. In stringent tests using training and validation datasets, the model exhibited outstanding predictive performance, with AUC values reaching 0.942 and 0.900, significantly surpassing other single-factor models. The nomogram model demonstrated satisfactory performance in predicting microvascular invasion in HCC. Looking back at previous studies, although nomogram models based on radiomics and clinical factors have shown promising predictive capabilities, most of them still have limitations in performance, with AUC values generally ranging from 0.801 to 0.861 (28–30). Notably, these models often involve numerous risk factors, whereas our nomogram model incorporates only three independent risk factors, highlighting the advantages of multi-omics approaches in data processing and model development.

In previous explorations, such as the study by Banerjee et al. (28), they delved into the radiogenomics of MVI in liver cancer, innovatively developing a novel imaging biomarker called radiogenomic venous invasion (RVI) by combining venous invasion genes in hepatocellular carcinoma with dynamic contrast-enhanced CT. This achievement has achieved significant results in predicting MVI and prognosis. Similarly, Taouli et al. (29) also conducted in-depth research on the imaging characteristics and genomic data of hepatocellular carcinoma, successfully identifying imaging features related to aggressive hepatocellular carcinoma genes through a combination of preoperative CT or MR examinations and transcriptomic analysis.

However, our study adopted a more unique and precise approach. We directly used miRNAs closely related to liver cancer as variables in logistic regression analysis, screening out miRNAs species independently associated with MVI in liver cancer through rigorous univariate and multivariate analysis. Subsequently, we combined these crucial miRNAs with radiomics and clinical radiological features to construct an efficient and accurate prediction model, achieving satisfactory preoperative prediction of MVI. This achievement provides a powerful tool or method for the accurate prediction of microvascular invasion in hepatocellular carcinoma, guiding clinical decision-making, optimizing treatment plans, and ultimately improving the survival rate and quality of life of patients.

### Limitations

This study still has some limitations. First, it is a small, single-center study. Therefore, the results should be complemented by further validation from larger queues at other centers. Second, miRNAs have many types, and only a small number was detected in this study. Secondly, there are many types of miRNAs, only a small amount was detected in this study, although this part of miRNAs has been shown to be associated with HCC caused by hepatitis B virus (HBV) (17), and the vast majority of patients we included are accompanied by HBV infection, but the effect of this data on HCC caused by non-HBV is unknown, so the results may only be valuable for HBV-associated HCC, in addition, whether there is genomic data with better performance than miR-21 needs to be further explored and verified. Third, miRNA is still a developing biomarker and is reported to have low reproducibility (30). Although we strictly follow standard procedures in the process of extracting miRNA, miRNA data stability is susceptible to a variety of factors such as limited amount of analyte before analysis, cell contamination, risk of inhibition, etc., which may introduce some bias into the final result. It is believed that with the development of liquid biopsy technology, the reproducibility and stability of miRNA data will be improved, so as to be used for robust clinical prediction. Fourth, MVI involves the tumor edge, but only the internal characteristics of the tumor were analyzed, and the ROI outside the tumor, especially around the tumor, was not expanded. In some studies (10, 31, 32), radiological features were extracted by expanding the ROI, achieving good results. This method is also a part of the follow-up research that needs to be further improved.

## Conclusion

The radiogenomic nomogram exhibited promising preoperative predictive capabilities and clinical decision-making implications in forecasting microvascular invasion (MVI) in hepatocellular carcinoma (HCC). This model holds the potential to emerge as a biomarker for MVI in HCC in the future, though its efficacy necessitates further validation through extensive studies encompassing larger sample sizes from multiple centers.

## Data availability statement

The data analyzed in this study is subject to the following licenses/restrictions: Not publicly available due to privacy of laboratory data. Requests to access these datasets should be directed to QZ, wfcyzhangqian@126.com.

## Ethics statement

The studies involving humans were approved by Institutional Review Board of Yantai Yuhuangding Hospital, Affiliated Hospital of Qingdao University. The studies were conducted in accordance with the local legislation and institutional requirements. The human samples used in this study were acquired from primarily isolated as part of your previous study for which ethical approval was obtained. Written informed consent for participation was not required from the participants or the participants’ legal guardians/next of kin in accordance with the national legislation and institutional requirements.

## Author contributions

GH: Formal Analysis, Investigation, Methodology, Writing – original draft. JQ: Data curation, Writing – review & editing. JG: Data curation, Writing – review & editing. YC: Data curation, Visualization, Writing – review & editing. FW: Data curation, Formal Analysis, Writing – review & editing. HZ: Writing – review & editing. HZ: Conceptualization, Data curation, Methodology, Writing – review & editing. XW: Data curation, Supervision, Writing – review & editing. HM: Investigation, Writing – review & editing. HX: Formal Analysis, Project administration, Writing – review & editing. CX: Supervision, Writing – review & editing. NL: Writing – review & editing, Software, Supervision. QZ: Funding acquisition, Supervision, Writing – review & editing.
