# Supplementary material for: Radiogenomics nomogram based on MRI and microRNAs to predict microvascular invasion of hepatocellular carcinoma
Source: Front Oncol. 2024 Jul 11;14:1371432. doi: 10.3389/fonc.2024.1371432 (PMC11269143; doi:10.3389/fonc.2024.1371432)
Supplement: Supplementary file 1 [file DataSheet_1.docx]

**Collection of plasma samples and** **miRNA extraction**

Using a sterile syringe, draw the subject's peripheral blood into an ethylene diamine tetracetic acid (EDTA) anticoagulant tube. Immediately mix it gently, then centrifuge at 2,000 rpm for 10 minutes at room temperature. Transfer 0.5–1 mL of the upper layer of clear plasma to a new centrifuge tube, then centrifuge at 16,000 rpm for 10 minutes. Finally, the supernatant was transferred to a clean storage tube at −80 °C for safekeeping and to avoid repeated freezing and thawing for more than five times (all the appliances used were RNA-exclusive, disposable consumables without RNase and DNase). Plasma miRNA was extracted in accordance with the instructions in the plasma miRNA rapid extraction kit [miRNeasy Serum/Plasma Kit (QIAGEN-217184)], and reverse transcription was performed with the PrimeScript RT Master Mix from TaKaRA. The qPCR reaction was performed using self-equipped PCR buffer from Wuhan Yzy Medical Science & Technology Co., Ltd. and Taq HS enzyme from TaKaRA. The reaction conditions were as follows: 95 °C for 5 min (95 °C for 15 s and 60 °C for 40 s for fluorescence collection) for 40 cycles. A fluorescence quantification system was used to analyze the relative expression of seven miRNAs (miR-122, miR-192, miR-21, miR-223, miR-26a, miR-27a, and miR-801) associated with hepatocellular carcinoma ^1^. Of the seven miRNAs measured in patient plasma, only five (miR-122, miR-21, miR-223, miR-26a, miR-27a) were ultimately included in the analysis due to the large difference in relative expression of miR-192 and miR-801 and the lack of stability of the data. The content of all miRNAs was expressed as a Ct (cycle threshold) value, and the lower the Ct value is, the higher the corresponding miRNA is expressed (vice versa).

**References:**

1. Zhou J, Yu L, Gao X, Hu J, Wang J, Dai Z, Wang JF, Zhang Z, Lu S, Huang X, Wang Z, Qiu S, Wang X, Yang G, Sun H, Tang Z, Wu Y, Zhu H, Fan J. Plasma microRNA panel to diagnose hepatitis B virus-related hepatocellular carcinoma. *J CLIN ONCOL* 2011; **29**: 4781-8.
