# Supplementary material for: Radiogenomics nomogram based on MRI and microRNAs to predict microvascular invasion of hepatocellular carcinoma
Source: Front Oncol. 2024 Jul 11;14:1371432. doi: 10.3389/fonc.2024.1371432 (PMC11269143; doi:10.3389/fonc.2024.1371432)
Supplement: Supplementary file 2 [file DataSheet_2.docx]

**MRI Examination**

The scan parameters were as follows: T1WI: TR of 4.0 ms, TE of 1.5 ms, FOV of 360 mm × 360 mm, layer thickness of 5 mm, and matrix of 256 × 192; T2WI: TR of 6667 ms, TE of 85 ms, FOV of 380 mm × 320 mm, matrix of 256 × 256, and layer thickness of 6 mm; DWI: TR of 8000 ms, TE of 69.8 ms, FOV of 400 mm × 320 mm, matrix of 380 × 380, layer thickness of 6 mm, and selected b-value of 800 s/mm^2^; DCE-MRI: TR of 4.5 ms, TE of 1.7 ms, FOV of 380 mm × 380 mm, layer thickness of 5 mm, and matrix of 256 × 192. DCE-MRI scans were performed using an autoinjector to inject the contrast agent Gd-DTPA at a flow rate of 2.5 mL/s after pre-scan with a use of 0.2 mL/kg of contrast agent and an equivalent saline rinse. After contrast injection was conducted, scans were performed at 30–35 (arterial phase, AP), 65–75 (portal phase, PP) and 180–300 s (delayed phase, DP) to obtain a three-phase image of the enhanced scan.
