# Supplementary material for: Radiogenomics nomogram based on MRI and microRNAs to predict microvascular invasion of hepatocellular carcinoma
Source: Front Oncol. 2024 Jul 11;14:1371432. doi: 10.3389/fonc.2024.1371432 (PMC11269143; doi:10.3389/fonc.2024.1371432)
Supplement: Supplementary file 3 [file DataSheet_3.docx]

**Analysis of Radiological Characteristics**

The qualitative radiological characteristics were assessed as follows: (a) maximum tumor length, defined as the largest diameter on the image; (b) tumor margin, classified as smooth and non-smooth edges on the images; (c) number, defined as the number of tumor divided into single and multiple; (d) enhancement pattern, classified as typical and atypical enhancement (typical enhancement is defined as tumor enhancement in the arterial phase and washout in the portal phase; the other modes are atypical); (e) radiologic capsule, defined as a highly enhancing margin that is wrapped around the tumor in the portal or delayed phase; (f) arterial peritumoral enhancement, defined as crescentic or polygonal enhancement around the tumor border detectable on arterial phase images; it becomes isointense in the delayed phase; and (g) intratumor hemorrhage or necrosis, defined as the presence of a bleeding signal or component without reinforcing.
